# Toward a rational and ethical sociotechnical system of autonomous vehicles: A novel application of multi-criteria decision analysis

**DOI:** 10.1371/journal.pone.0256224

**Published:** 2021-08-13

**Authors:** Veljko Dubljevic, George List, Jovan Milojevich, Nirav Ajmeri, William A. Bauer, Munindar P. Singh, Eleni Bardaka, Thomas A. Birkland, Charles H. W. Edwards, Roger C. Mayer, Ioan Muntean, Thomas M. Powers, Hesham A. Rakha, Vance A. Ricks, M. Shoaib Samandar

**Affiliations:** 1 North Carolina State University, Raleigh, NC, United States of America; 2 Oklahoma State University, Stillwater, Oklahoma, United States of America; 3 University of North Carolina, Chapel Hill, NC, United States of America; 4 University of North Carolina, Asheville, NC, United States of America; 5 University of Delaware, Newark, Delaware, United States of America; 6 Virginia Polytechnic Institute and State University, Blacksburg, Virginia, United States of America; 7 Guilford College, Greensboro, NC, United States of America; Tsinghua University, CHINA

## Abstract

The impacts of autonomous vehicles (AV) are widely anticipated to be socially, economically, and ethically significant. A reliable assessment of the harms and benefits of their large-scale deployment requires a multi-disciplinary approach. To that end, we employed Multi-Criteria Decision Analysis to make such an assessment. We obtained opinions from 19 disciplinary experts to assess the significance of 13 potential harms and eight potential benefits that might arise under four deployments schemes. Specifically, we considered: (1) the status quo, i.e., no AVs are deployed; (2) unfettered assimilation, i.e., no regulatory control would be exercised and commercial entities would “push” the development and deployment; (3) regulated introduction, i.e., regulatory control would be applied and either private individuals or commercial fleet operators could own the AVs; and (4) fleets only, i.e., regulatory control would be applied and only commercial fleet operators could own the AVs. Our results suggest that two of these scenarios, (3) and (4), namely regulated privately-owned introduction or fleet ownership or autonomous vehicles would be less likely to cause harm than either the status quo or the unfettered options.

## 1. Introduction: Multi-criteria decision analysis and the problems of autonomous vehicles

The introduction of devices and systems that capitalize on artificial intelligence (AI) and autonomous systems has shown the potential to generate enormous social good [1, 2]. However, there are also serious ethical and safety concerns [3–6]. Transportation is one of the domains in which AI technology is increasingly adopted [7]. As autonomous vehicles (AVs) are implemented in various types of transportation systems, the degree of direct interaction between AI-controlled vehicles and humans (e.g., pedestrians) and human operated vehicles [connected non-autonomous vehicles (CVs) and traditional vehicles] will grow. Therefore, controlling the behavior of the AVs becomes inherently more complex, and the potential for harm to humans increases. In the current realizations of AVs, it is possible to have vehicles control their trajectories in simple situations, like single lanes, precisely carrying out the instructions of the owners, according to relatively simple programming. However, in complex settings, where the interactions are far more complicated, humans frequently take actions outside the bounds of a nominal rule set to resolve conflicts. Therefore, successfully implementation of AVs will require accommodating unpredictable situations that may occur as a result of human behavior and decision-making.

The successful implementation of AVs is not only an engineering issue but a social, political, and ethical one as well. The perspectives of multiple disciplines are required to craft holistic assessments of the impacts of AVs in different types of controlled and uncontrolled transportation settings. Understanding the societal and ethical implications of AVs (and more generally, any AI system) inherently involves many distinct issues: the nature and capabilities of the technologies employed (computer science, engineering), how humans can and should use them (ethics), how humans will behave in response to the presence of AVs in the traffic stream (social sciences), and the technology’s impact on socio-economic structures (political science, economics). Thus, producing new and relevant knowledge in this area requires the expertise originating in multiple disciplines [8].

Multi-Criteria Decision Analysis (MCDA) is a method by which it is possible to study potential harms and risks [9–13]. The basic tenet is that MCDA, along with qualitative techniques, can provide defensible insights about the way people see the multi-faceted impacts of technological change. Since the first introduction of MCDA, studies have 1) expanded the number of criteria that can be considered [12], 2) the ways to capture the relative importance (or weights) of different harms [10], 3) the techniques for comparing harm/benefit ratios [13], and 4) ways to make clear the perceptions of all relevant stakeholders [14].

One of the strengths of MCDA is that it can capture expansive areas of knowledge in a transparent manner, allowing for replication and improvement of the methodology [14]. MCDA breaks down complex evaluations into a series of smaller, more easily assessed issues, thus enhancing the reliability and validity of the results.

We perceive there is a pressing need to address relevant social concerns so that the development of AI-empowered systems account for ethical standards. Doing so will facilitate the responsible integration of these systems in society. To this end, we use the Delphi method and a consensus workshop as forms of input to develop a formal Multi-Attribute Impact Assessment (MAIA) questionnaire, which then enables us to use MCDA examining the social and ethical issues associated with the uptake of AI. We have focused on the domain of AVs because of their familiarity and imminent introduction [15]. However, the AVs serve as a stand-in for the broad range of domains in which intelligent, autonomous agents will, in the future, interact with humans, either on an individual level (e.g., pedestrians, passengers) or a societal level [16].

By utilizing both qualitative and quantitative analyses, we have expanded the utility of MCDA, giving it the potential to drastically improve the ethical evaluations of transformative change, illustrated here in the context of AV technology. The MAIA questionnaire provides an evidence base regarding percieved impacts, including the raw data for harm-over-benefit ratio analyses. Notably, our approach addresses the drawbacks identified in the literature critical of the MCDA methodology, such as lack of attention to situational factors [17], value judgments [18], and additional stakeholders [19–21].

Expert opinions are periodically obtained on emerging technologies to provide valuable insights [22–24]. However, a comprehensive methodology for comparing heterogeneous harms and benefits from the perspective of different stakeholders has been lacking. Previously, expert assessments of AV technology have been based on fictional future scenarios so that possible policies could be discussed [25], as opposed to identifying how policies adopted in the present could shape the future, or how proposed policy options compare to the present one in terms of relevant criteria (see Table 1).

**Table 1:** The harms and benefits assessed.

Q1	Harms of vehicle related mortality (e.g., driver or passenger deaths on the road)
Q2	Harms of vehicle specific damage (e.g., costs of damage to property)
Q3	Harms of vehicle related damage (e.g., damage to natural environment)
Q4	Harms of vehicle system encroachment on human living (e.g, reduction of urban walkability)
Q5	Harms of vehicle related occupational injuries (e.g., sedentary lifestyle of drivers)
Q6	Harms of vehicle related lack of status (e.g., elderly losing driver’s licenses due to visual impairments)
Q7	Harms of vehicle related loss of time or productivity (e.g, time spent in traffic jams)
Q8	Harms of vehicle related loss of social engagement (e.g., time spent isolated from others)
Q9	Harms of vehicle related injury to others (e.g., hit and run incidents)
Q10	Harms of vehicle related economic costs (e.g., maintenance costs)
Q11	Harms of vehicle related changes to community (e.g., marginalization of specific communities)
Q12	Harms of vehicle related crime opportunities (e.g., sexual assault by ride-hailing service drivers or passengers)
Q13	Harms of vehicle related economic changes (e.g., loss of jobs by drivers)
Q14	Benefits of promoting societal value (e.g., increase in economic activity)
Q15	Benefits of minimizing negative societal impacts (e.g., decrease in pedestrian injury and death)
Q16	Protecting the interests of users (e.g., drivers)
Q17	Advancing the preservation of the environment (e.g., reducing traffic jams)
Q18	Maximizing the progress of science and technology (e.g., increasing data quality)
Q19	Engaging relevant communities (e.g., pedestrians, business communities)
Q20	Ensuring oversight and accountability (e.g., preventing or limiting irresponsible uses)
Q21	Recognizing appropriate governmental and policy roles (e.g., bringing public attention to transportation issues)

We fill this gap by eliciting expert opinions about the impacts of AVs under several realistic adoption scenarios through a Delphi exercise [26] and a consensus workshop [27]. We then use MCDA to conduct a formal analysis, resulting in operational evidence regarding the moral, social, and economic benefits and harms of AVs. Our identification of relevant facts and values—the task for which disciplinary experts are essential—helps us conduct a complex evaluation, reduce confounds and biases, and clarify uncertainties [28].

Our assumption is that for the foreseeable future AVs will not completely replace traditional non-autonomous motor vehicles. We expect that AVs will operate in a heterogeneous environment alongside traditional vehicles, as well as cyclists and pedestrians. Existing vehicle technology is assumed to be robust and desirable to preserve not merely for economic reasons but also for psychological ones, such as the ‘joy of driving’ [29]. Thus, we agree with Samandar and colleagues that “a mixed traffic fleet is likely to be the predominant scenario for the foreseeable future.” [30]

Studies similar to ours, in other countries, have generated assessments that are interesting but not necessarily applicable to the U.S. context. For instance, the German Federal Ministry of Transport and Digital Infrastructure appointed a national ethics committee for automated and connected driving to develop and issue a code of ethics. This code states that “protection of individuals takes precedence over all utilitarian considerations” and “automated driving is justifiable only to the extent to which conceivable attacks, in particular manipulation of the IT system or innate system weaknesses, do not result in such harm as to lastingly shatter people’s confidence in road transport.” [31] Such guidance is interesting, but there is no mention of how it is to be implemented, raising concerns of its feasibility. Moreover, the policy fails to address important issues such as how AV technology could be programmed to resist malicious actors, such as terrorists [32, 33], or how social justice issues can be safeguarded during the introduction of AVs into the socioeconomic system [34]. The European Union [24] and Australia [35] have also developed expert-based scenarios intended to guide policy makers in regulating AV technology. Groups of experts are very important, but they are better used in assessing the importance of harms and benefits, as we have done.

## 2. Materials and methods

We develop a novel instrument for the application of the MCDA method, which we call the Multi-Attribute Impact Assessment (MAIA) questionnaire, to assess the impacts of AV technology. We identified 21 impacts for which we sought expert opinions about their importance. We followed an iterative process that began with the first author of this paper preparing an initial list of harms and benefits based on the AV ethics literature and relevant agency reports [36, 37]. This list was discussed at length by a sample of other experts (the first six authors of this paper), and then revised based on the feedback. It was subsequently piloted in a Delphi survey with the full panel of 19 experts, again revised based on feedback and then discussed at length during the consensus workshop (see below). The final list of impacts was categorized into 13 harms and eight benefits, as shown in Table 1.

Concurrently, we explored four operational scenarios or regulatory environments under which AVs might be introduced. They are described in Table 2. Our co-authors with expertise in the AV domain suggested a set of feasible (ideal-typical) options based on their extensive familiarity with the technology, whereas the group as a whole estimated the impact and consequences of these options [26].

**Table 2:** Operational scenarios and regulatory environments explored.

#	Definition	Description
1	Status Quo (S-Q)	The transportation system as it is currently, with non-AVs.
2	Unfettered AVs (U-F)	A transportation system in which there is no regulation and so implementation is unfettered and left to commercial entities (i.e., the tech industry).
3	Regulated privately owned AVs (R-P)	A transportation system which is regulated so that AVs are owned much like traditional passenger vehicles. They must be inspected and there are only certain “areas” where they can be operated.
4	Regulated fleet owned AVs (R-F)	A transportation system which is regulated so that AVs are owned only by commercial fleets, with stringent inspections, and there are designated areas where they can be operated.

Note: In scenarios 2–4, we assume that traditional non-autonomous vehicles continue to operate in addition to AVs.

Beyond the *status quo* (scenario 1), the first AV condition (scenario 2) assumes no regulatory control will be exercised and commercial entities will “push” the development and deployment. Implicitly, anyone (any entity) would be able to purchase and operate such vehicles anywhere.

The second and third AV conditions (scenarios 3&4) assume that regulatory control will be imposed. In Scenario 3 (private) individuals will be able to purchase and operate AVs. Scenario 4, on the other hand, assumes only fleet operators will be able to purchase and operate AVs. Scenarios 3 and 4 assume SAE level 4, meaning that the vehicles can operate on a portion of the highway network [37]. In scenario 3, companies can also own AVs, like car rental and ride sharing companies, but there is no prohibition against people having them. In scenario 4 only commercial operators can own AVs; no personal ownership is allowed. The scenarios are silent about market penetration; but implicitly, they assume the AV population is large enough that their operational impact is visible. We elected not to focus on or assume SAE Level 5, which is full autonomy, as one of the scenarios because it seems far off in the future compared with the status quo.

As noted above, the workshop included 19 leading researchers. They were from diverse backgrounds in terms of discipline, including political science/public policy, civil/transportation engineering, philosophy/ethics, computer science/AI, organizational behavior. They also were diverse in terms of gender and ethnicity, including people with African-American, Asian-American, Caucasian and immigrant backgrounds. They participated in a consensus-building workshop on the NC State campus on 21 Feb 2020. We selected 19 participants because that cohort is near the upper limit espoused by Phillips [27] for effectiveness in expert-based decision analysis studies. Most of the participants are co-authors of the paper. (The four experts who thought that their contribution did not rise to the level of authorship are listed in the acknowledgements). The participants discussed the criteria and the scenarios at length during the workshop. Five Qualtrics surveys were administered, eliciting input from the participants: 1) weights among the criteria, 2) a 4-point assessment of harms, 3) a 4-point assessment of benefits, 4) a 10-point assessment of harms, and 5) a 10-point assessment of benefits. After the workshop, an additional survey of weights limited to 100% total for all criteria was conducted.

The Delphi method was used to generate the first and last wave of responses, programmed in Qualtrics. During the consensus workshop, the participants were briefed on the results of the first survey and then the additional surveys were administered. The responses were converted to a 10-point scale, the participants were briefed about the results, and the survey was repeated a second and third time. The repetition of rankings (using a 4-point scale and a 10-point scale) helped reduce potential biases in the impact assessments.

The expert input during the Delphi surveys and consensus workshop was further analyzed with the aid of an MCDA software. The software used an R package from Diviz and Qualtrics surveys as input. The data was cleaned and processed before performing the weighted sum MCDA and plots were created showing the results. The software and raw data are available at the GitHub repository: https://github.com/niravajmeri/RISF-MCDA-Diviz.

## 3. Results

**Fig 1:**
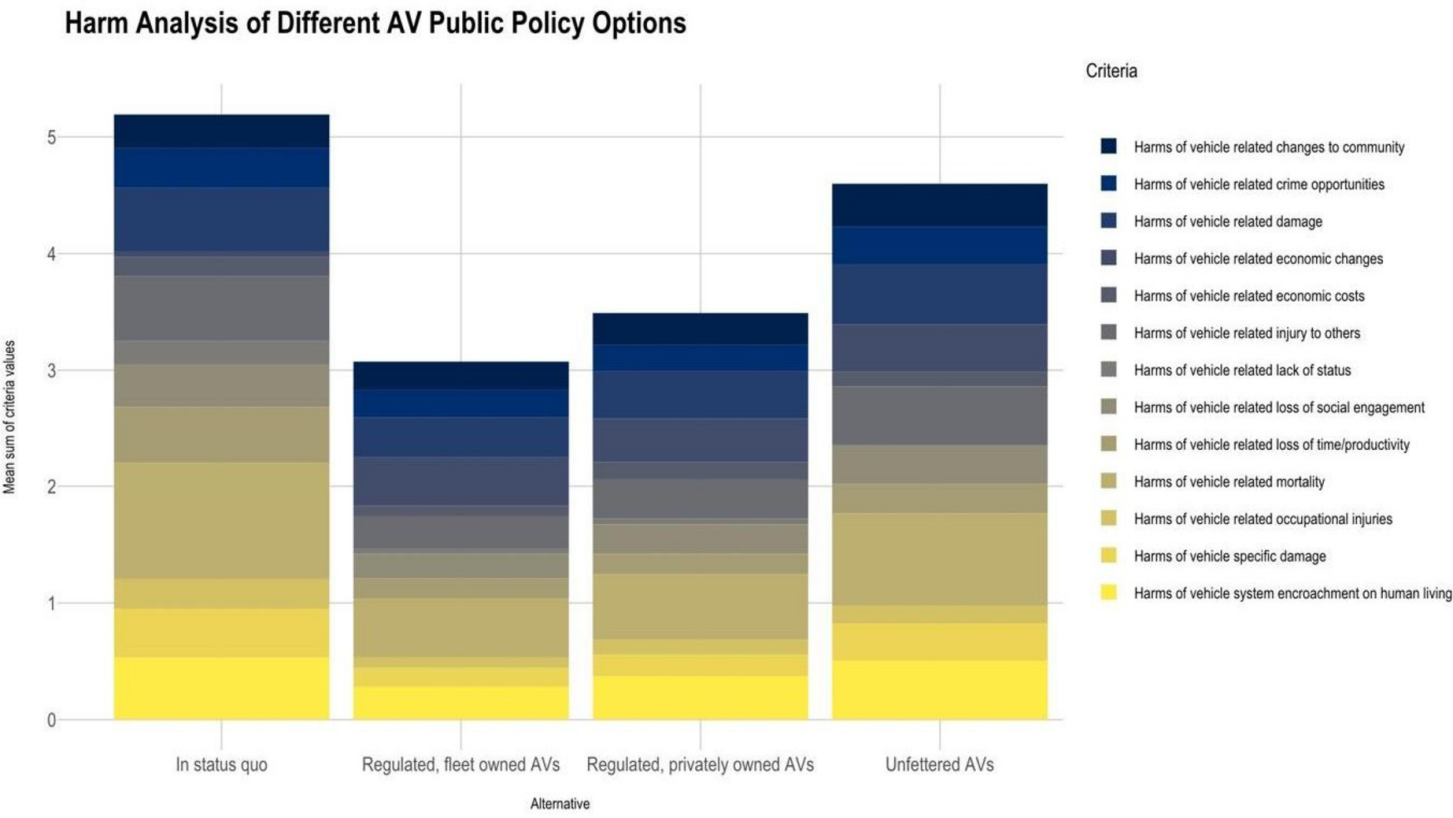
Harms of different AV technology implementation, 4-point scale.

A consensus emerged that certain forms of AV implementation would be less harmful than others. Namely, the regulated private or fleet owned scenarios (3 or 4) would be better than either the status quo (scenario 1) or the haphazard or unfettered scenario (2). The stacked histograms in Fig 1, which show the harms of different AV technology implementation measured on a 4-point scale, summarize this finding.

**Fig 2:**
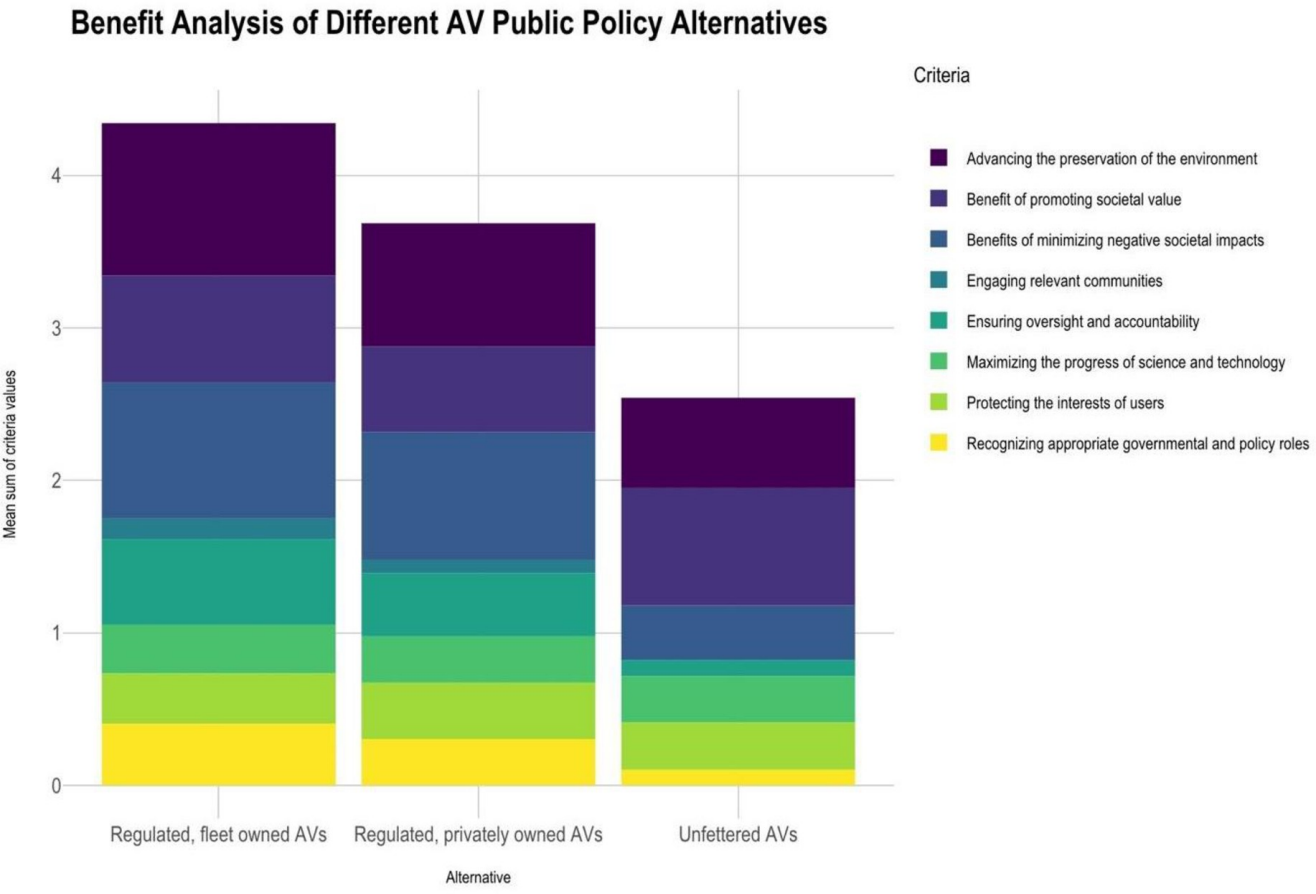
Benefits of different AV technology implementation, 4-point scale.

**Fig 3:**
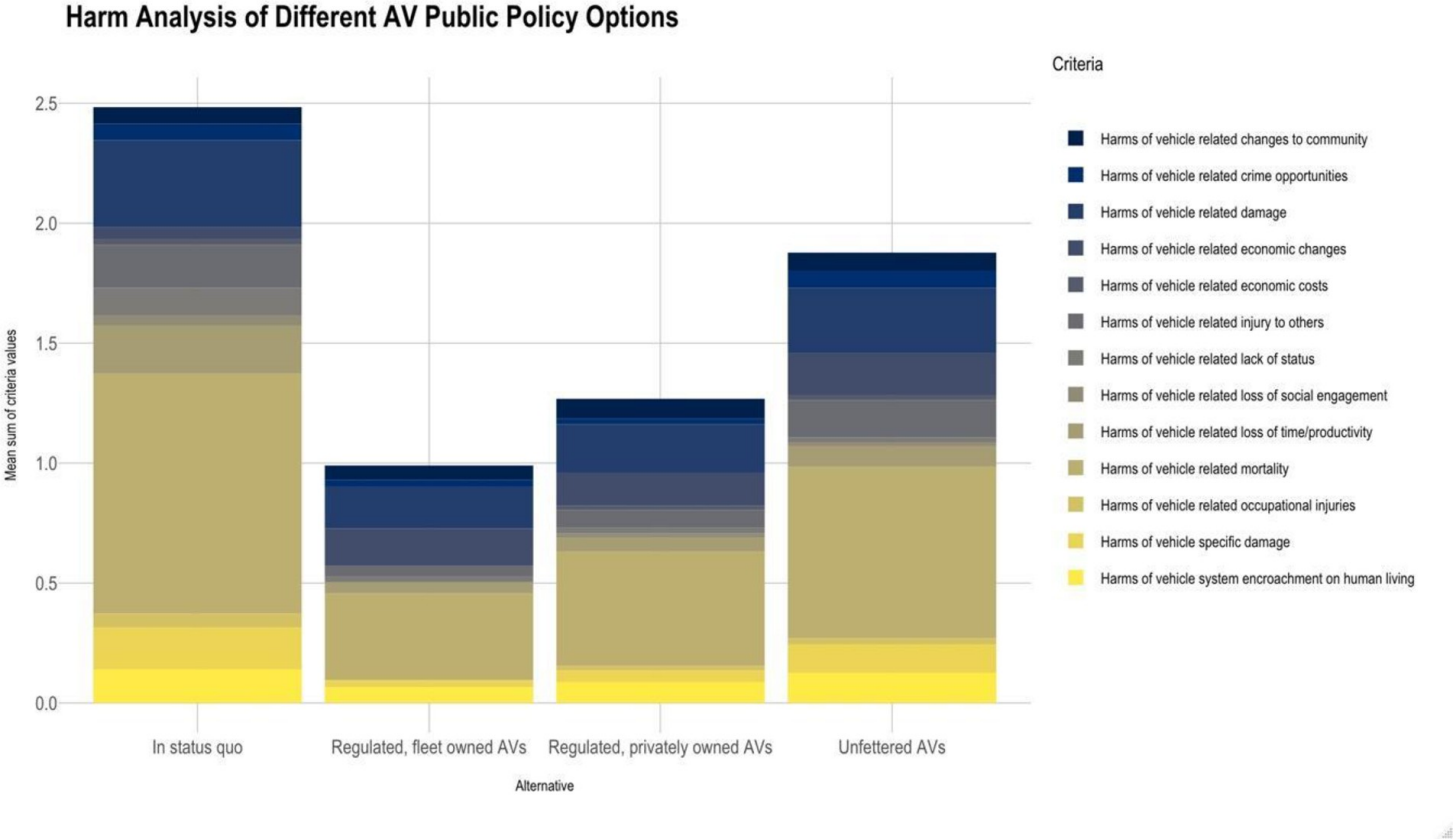
Harms and benefits in a repeated, 10-point scale.

Similarly, the regulated, fleet owned scenario (4) was perceived to produce the greatest benefits (see Fig 2). A follow-up survey that used a 10-point scale produced similar results. See Fig 3.

**Fig 4:**
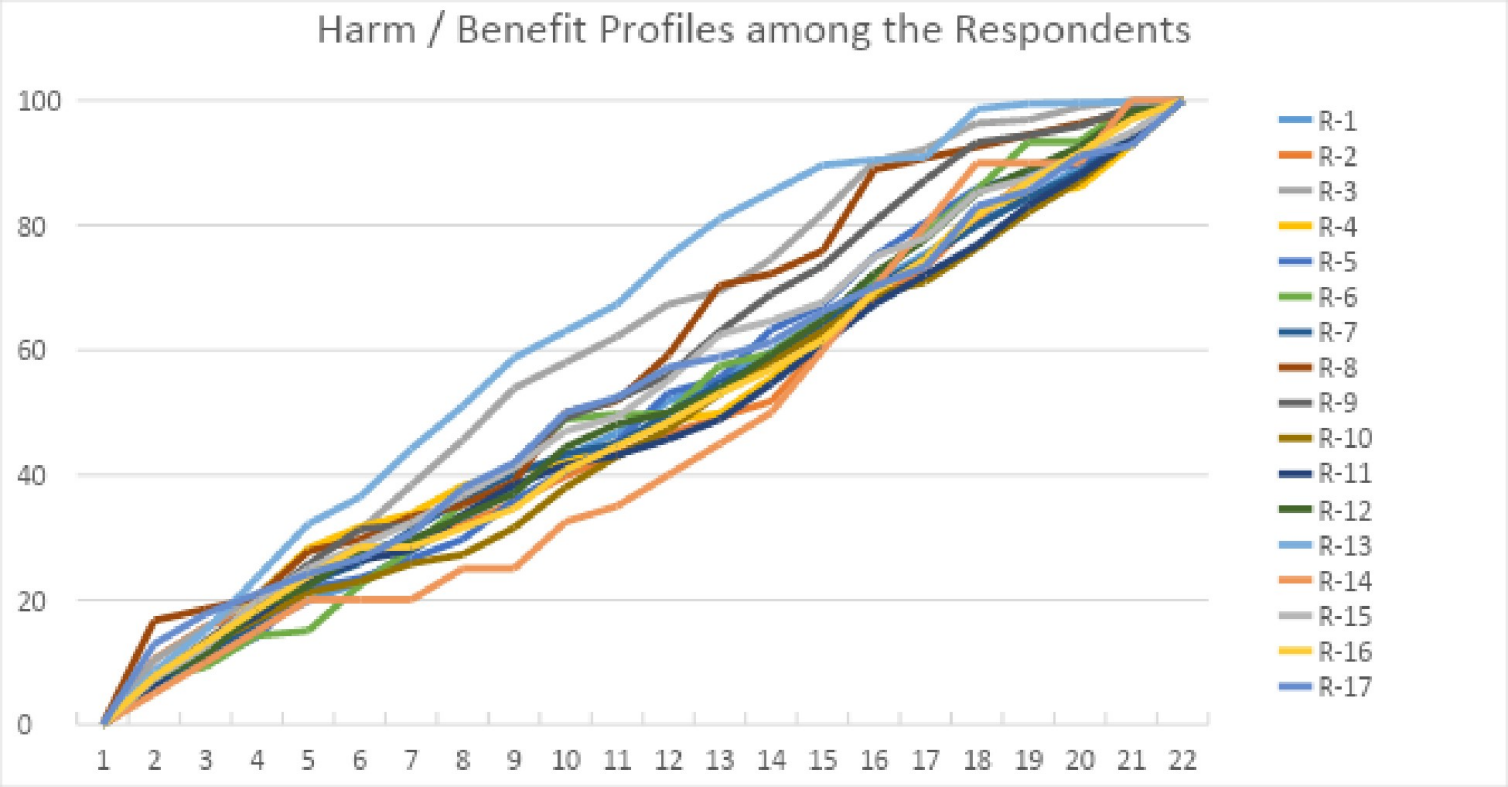
A CDF-like display of the harm / benefit assessments.

The harm and benefit assessments were open ended. That is, the respondents were allowed to scale their total assessments on any basis. To make this clear, whereas one respondent could have used “1” as the maximum for each, another could have used “100”. With 21 criteria, this means the first respondent would have had a harm and benefit assessment totaling up to 21; the second would have had a maximum total of 2100. We chose this strategy because we wanted to see if the respondents would provide similar assessments of the relative importance of the 21 criteria. Then based on the total number of points they provided, we scaled their assessments to a total of 100. Fig 4 shows the “weighted profiles” that emerged. For each respondent, the profile shows the percentage distribution of importance among the 21 criteria for a given respondent. To help the reader understand these profiles, two hypothetical examples are useful. First, if a respondent had indicated that all harms and benefits were of equal value, the “profile” would have been a straight line. We did not see one of these. Second, if a respondent had put more points on some and less on others, the “quick rises” in the profile would be associated with criteria they deemed important, the “slow rises”, those they deemed less important. The main conclusion we draw from this figure is that, except for a couple of respondents, all of the participants had a similar sense of the relative importance of the harms and benefits. Respondent 5 (medium blue and the highest) gave the greatest aggregate importance to the harms (highest total percent by impact 13). Respondent 14 (dark orange, and the lowest) gave the greatest importance to the benefits (lowest total percent by impact 13).

### 3.1. Harms

Two “harm” assessment surveys were administered. The harms were impacts 1–13. One survey used a 4-point scale (0–3) for each impact where zero was “no harm” and 3 was “extreme harm.” The other used a 10-point scale where 1 was “no harm” and 10 was “extreme harm”. The assessments were done sequentially, with the 4-point scale being used first. Since the findings from the 4-point scale were shared with the participants before the 10-point survey was administered, the results from the 10-pomt survey have been informed by the 4-point one.

We analyzed the harm responses in several ways. The first was in terms of the relative importance of the harms; another was by “scenario.” The description of individual harms is presented as in Table 1 (questions 1–13); and the four scenarios, in Table 2.

**Fig 5:**
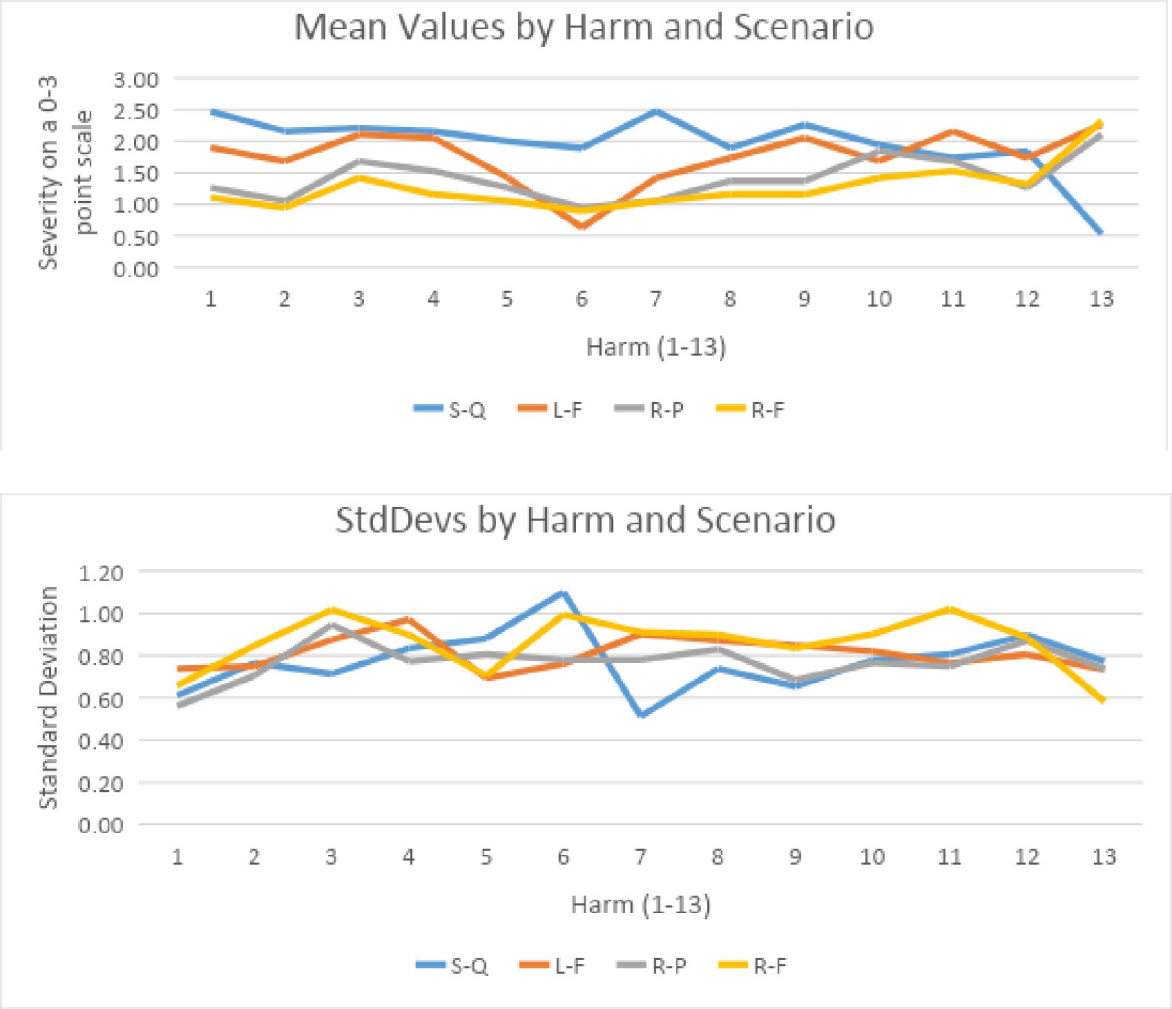
Means and standard deviations for 4-point assessments (0–3) by harm and scenario.

The mean values and standard deviations based on the 4-point scale (0–3) are shown in Fig 5. A higher score means a greater harm was perceived. The harm with the greatest reduction due to AVs is 6, it was termed “lack of status loss” which could also be thought of as “status preservation” (e.g., a person’s mobility is not diminished due to visual impairment). This makes sense; AVs provide a significant boost in mobility for these people. The one where the impacts are mixed or minimal is 11, harms related to changes to community (e.g., the marginalization of specific communities). The respondents saw no clear trend in this impact. The impact with the greatest variation in impact assessment was 3, damage caused by the vehicles to the natural environment. We suppose this is because of differences in perceptions about the technology and how it will be used. Harm 13 stands out as having characteristics different from the others. It pertains to economic changes caused by the AVs (e.g., loss of jobs by drivers). Hence, it is not surprising that its impacts are different. The aggregate assessment of differences among scenarios will be addressed later, but it seems clear that scenario 1 has the greatest harms, followed by scenarios 2, 3, and 4, roughly in that order. Scenario 4, which involves a regulated commercially owned fleet, has the greatest reduction in harms.

**Fig 6:**
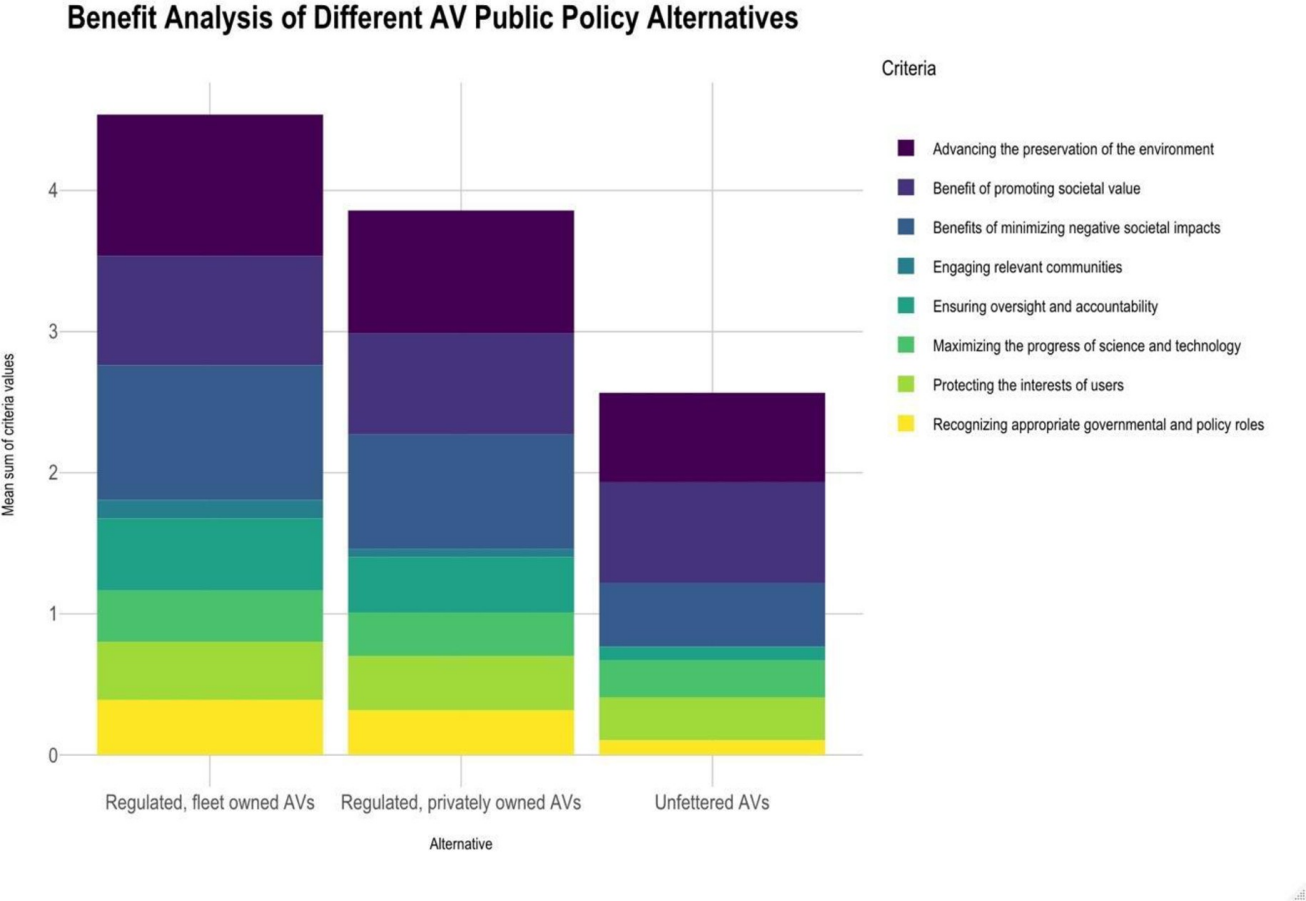
Mean values and standard deviations for 10-point assessments (0–9) by harm and scenario.

Fig 6 shows the same information but on a 10-point scale. The 1–10 results were remapped to 0–9 so that the low end of both assessments was 0. Strikingly different are the assessments for criterion 1 (more spread) and 3 (a higher sense of harm for the status quo). Otherwise, the pattern is similar. Moreover, as before Scenario 1 has the greatest harms, followed by Scenarios 2, 3, and 4, roughly in that order.

**Fig 7:**
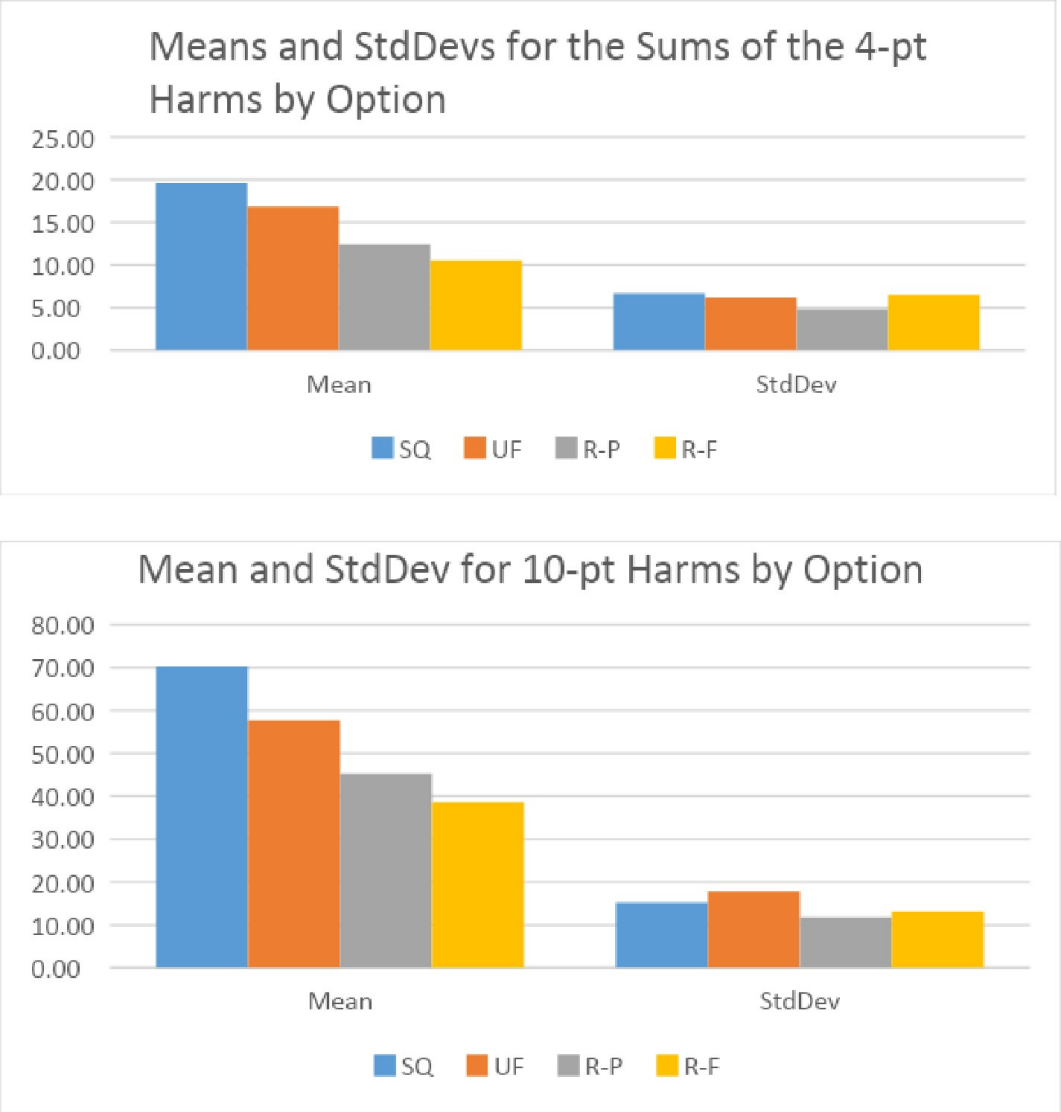
Means and standard deviations for the sums of the harms (by respondent) based on 4-point and 10-point ratings.

For a broader brush, we computed the sums by respondent for all the harms (the sum of the responses to questions 1–13). A maximum of 52 (13*4) was possible; and a minimum of 0. We then computed the average of these values and the standard deviation. Fig 7 shows the results for both the four-point scale (0–3) and the ten-point scale (0–9). The trends in the average among the four scenarios is the same in both cases. The greatest harms are associated with the status quo (scenario 1); and the least with the regulated / fleet owned scenario (scenario 4). These findings are consistent with visual inspections of Figs 4 and 5. One noticeable difference is that the spread between the scenarios is larger in the 10-point case than in the 4-point instance. The trends in the standard deviations are also similar except that, in the case of the ten-point scale, the standard deviation for the laissez faire scenario (2) is higher than it is for the other three scenarios, whereas in the four-point assessment it is similar to the others. This could be an impact of the shared feedback on the 4-point survey.

### 3.2. Benefits

Two “benefit” surveys were administered. As with the harms, one used a 4-point scale (0–3) where zero was “no benefit” and 3 was “drastic benefits”; the other used a 10-point scale where 1 was “no benefit” and 10 was “drastic benefits.” For purposes of the presentation here, the 10-point scale has been re-scaled to 0–9 so that “0” is common between the two surveys. For the “status quo” scenario, the benefits were not assessed as it was assumed that the status quo would be the baseline.

**Fig 8:**
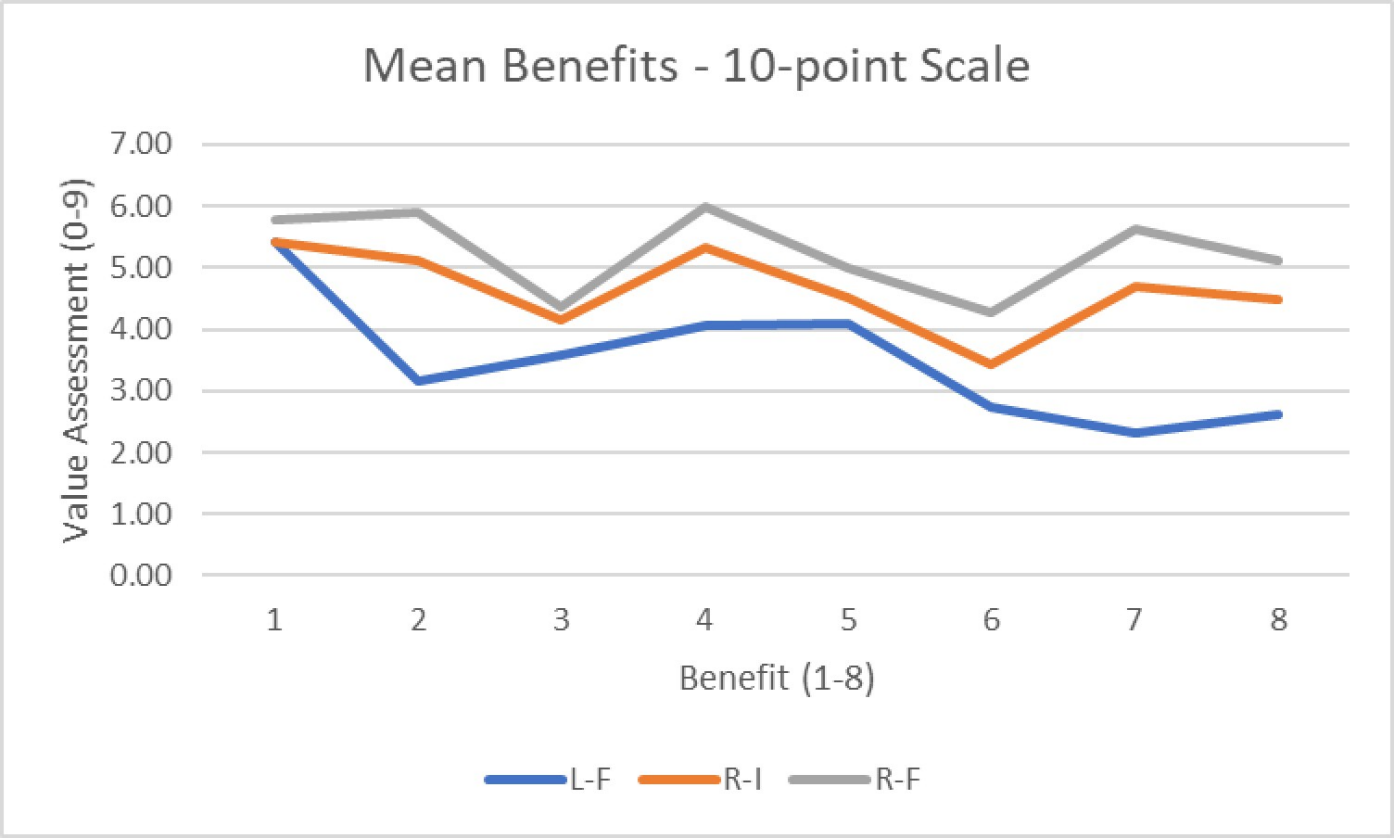
Average benefit value assessments for the 10-point scale.

Fig 8 shows the benefit assessments based on 10-point scale. Benefit 1 maps to impact or question 14 and benefit 8 to impact or question 21 as listed in Table 1. We find that the greatest benefits are associated with impacts 4 and 7, i.e., advancing the preservation of the environment (e.g., reducing traffic jams) and ensuring oversight and accountability (e.g., preventing or limiting irresponsible uses), respectively. These benefits are greater for the regulated scenarios (3 and 4) than for the unregulated one (2). Moreover, in a broader sense, the benefits of scenario 4 (regulated / fleet owned) are the greatest followed by scenario 3 (regulated / privately owned) and then scenario 2 (laissez faire or unfettered). The one benefit where scenario 2 produces comparable or higher benefits is the first one, promoting societal value (e.g., increase in economic activity). Intuitively, respondents perceived that deregulated development would produce the most innovation and capital investment.

### 3.3. Overall assessment

The summative question is this: does our study suggest a “best” scenario, weighing the harms and benefits? The answer seems to be “yes”, although there are many ways to answer the question in detail [38]. One possible approach is to take the harm and benefit value assessments, by respondent, and combine them with the corresponding weights (by respondent), then we obtain sums of the results for the harms and the benefits. Admittedly, this is “problematic” in that the weights for the harms and benefits were assessed together; and here, they have been normalized to sum to one. But that may not be “bad” or “wrong.” It can be argued that forcing them to sum to 1 provides, implicitly, the respondent’s sense of the relative value of the eight benefits versus the 13 harms. Further surveying will reveal valuable information about this issue.

**Fig 9:**
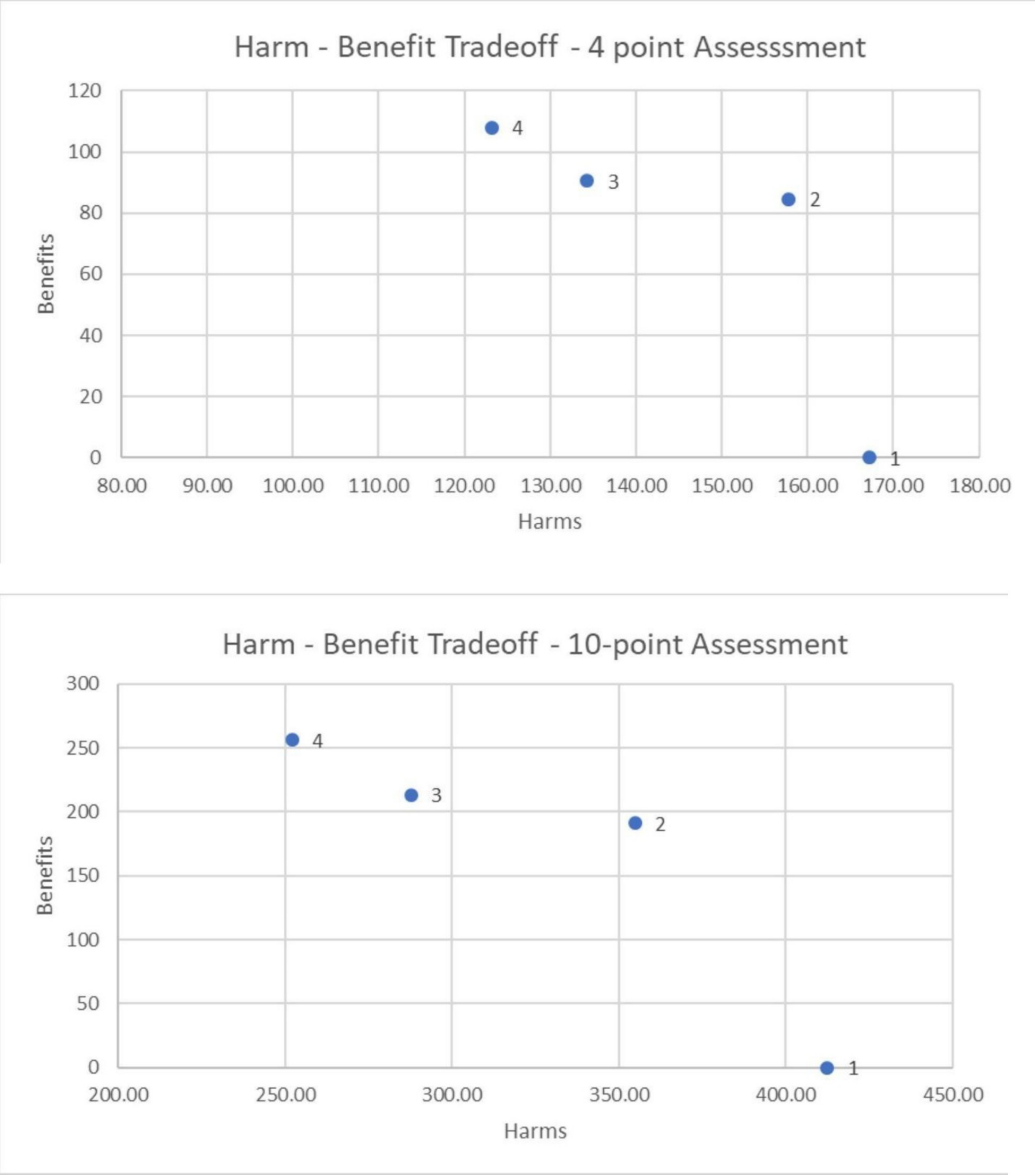
Harm/benefit tradeoffs for both the 4-point and 10-point assessments.

In this instance the “total of the weighted harms” has been plotted against the “total of the weighted benefits” for the four scenarios, based on the “weighted value assessments” of the respondents. Fig 9 plots the sum of these “weighted value assessments” for the harms against the “weighted value assessments” for the benefits. The message seems clear. The regulated-fleet owned scenario (4) seems to have greater benefits and lesser harms among the four. It is slightly better than the regulated-personally owned scenario (3) and clearly better than the *laissez-faire* or unfettered scenario (2). This is especially true for harms. (Of course, the status quo scenario has no benefits, and its harms are perceived to be the largest, significantly so in the case of the 10-point based assessment).

## 4. Discussion

Without intending to be negative, the introduction of AVs exposes society to an array of new risks. Despite the excitement surrounding this technology, there are many unanswered questions about whether it will be both beneficial and safe. Even though there are expectations of overall benefits to society from the deployment of AVs, some socio-economic groups compared to others could experience (much) higher costs relative to benefits [39]. These negatively affected groups are likely to include those whose livelihood depends on traditional motor vehicles. A significant number of drivers (and, by extension, family members depending on their economic activity) will be affected by the introduction of AVs. In this regard, there are approximately 1.7 million truck drivers in the United States [40], with about 800,000 involved in truck transportation. An exacerbating factor, potentially, is that there is currently a shortage of truck drivers [41]. This could drive even more the motivation to get AV trucks on the road promptly, thus pushing current professional drivers out of jobs sooner. Although AVs will create new jobs in the trucking industry and other industries [22], it is questionable whether these new jobs would outnumber those lost due to the AVs; indeed, that seems unlikely. However, many driving jobs are perceived to be unsatisfying and potentially unhealthy (e.g., due to high incidence of sleep apnea and obesity), making their eradication an overall positive outcome if other employment opportunities are available [35].

Positive or negative impacts [42] related to the changes wrought to society needed to be assessed in a free and open discussion by a multi-disciplinary panel of experts. Our results point to the need to better address how the public views the trade-offs between (1) safety; (2) physical ecology (environmental issues); (3) social ecology; (4) economic issues; and finally (5) the specific impacts for groups that will be most affected by AV implementation (e.g., professional drivers).

We contend that it is essential to increase the public’s confidence that the values of a pluralistic society are accounted for in the development of AV policies. This can be accomplished by 1) bringing society into the identification of norms surrounding AVs [43, 44] and 2) accounting for multiple elements of moral decision-making [45]. Regarding point 1, although expert groups like the one we assembled do not nearly represent society as a whole, if such a group is large enough and selected carefully it does represent an important slice of society that policymakers should pay attention to. Regarding point 2, such an expert group brings diverse, refined perspectives to moral decision-making that can only increase the reliability of the assessment by ensuring the most important considerations and values are brought to the surface.

Several states in the U.S. have started the process of legislating AVs, most notably designating the manufacturer of a vehicle operated by an automated driving system as the vehicle’s sole driver, and limiting this special legal framework to motor vehicle manufacturers that deploy their vehicles as part of fleets within specific geographic areas [46]. Our work provides valuable data that should inform policy makers of concerns and potential benefits of AV technology in specific implementation strategies, and this could improve the quality of the democratic policymaking process. We recommend that state legislatures and the federal government strongly consider incorporating our results regarding technology development scenarios as well as the MAIA questionnaire into their deliberations about the impact of AVs.

We endeavored to contribute a quantifiable estimation of three feasible policy options to the implementation of Autonomous Vehicles (AVs), which may (or may not) be adopted in different jurisdictions. As with any policy analysis work, we do not presume that we have made a clear-cut (to all concerned) resolution of the social and ethical issues arising from AVs, but we have provided a *prediction* [47] that one type of policy (i.e., regulated, fleet owned AVs), if implemented, would result in less harm and more benefit to society. We have also provided a questionnaire, the Multi-Attribute Impact Assessment (MAIA) as a possible instrument for measuring benefits and harms of differential policy implementation. Only time can tell if our prediction or our instrument (i.e., MAIA) turns out to be useful.
